# A revision of the *Cautires obsoletus* species group from Java (Coleoptera, Lycidae)

**DOI:** 10.3897/zookeys.241.3089

**Published:** 2012-11-13

**Authors:** Ladislav Bocak

**Affiliations:** 1 Department of Zoology, Faculty of Science, Palacky University, tr. Svobody 26, 771 46 Olomouc, Czech Republic

**Keywords:** Systematics, Metriorrhynchini, Indonesia, new species, new synonym

## Abstract

The Javanese fauna of the species group *Cautires obsoletus* is revised. Altogether, eight Javanese species were classified in the group; five of them are proposed to be junior subjective synonyms: *Cautires fruhstorferi* Dudkova & Bocak, 2010, a replacement name of *Bulenides lineatus* Pic, 1921, *Cautires javanicus* Bourgeois, 1883, *Cautires inhumeralis* (Pic, 1921), *Cautires nigromaculatus* (Pic, 1925), and *Cautires pudicus* (Kleine, 1931) (all synonymized to *Cautires obsoletus* Waterhouse, 1879). Three Javanese species are redescribed: *Cautires apicalis* (Pic, 1925), *Cautires obsoletus* (Waterhouse, 1878), and *Cautires singularithorax* (Pic, 1925). *Cautires apicalis* (Pic, 1925) is removed from the synonymy of *Cautires corporaali* (Pic, 1921) and reinstated as a valid name. Three new species are proposed: *Cautires walteri* **sp. n.**, *Cautires taoi* **sp. n.**, and *Cautires sukosarensis* **sp. n.** All species are keyed and principal diagnostic characters are illustrated. The distribution and relationships to *Cautires* fauna of other Great Sundas islands are briefly discussed.

## Introduction

The species of the *Cautires obsoletus* group were originally placed in *Bulenides* Waterhouse, 1879 (Kleine 1926, 1933, Bocak 2002, Bocak and Bocakova 2008). Although *Bulenides* was easily recognizable by the presence of a single areola in the pronotum and nine longitudinal costae in the elytra, a recent study showed that the genus is a polyphyletic assemblage of two independent lineages nested in *Cautires* Waterhouse, 1879 (Dudkova and Bocak 2010). One of these lineages is the *Cautires obsoletus* group characterized by an almost triangular shape of the pronotum and a long, slender phallus. The classification of Javanese species has, for a long time, been chaotic due to the inadequate work of M. Pic and lack of communication among taxonomists working on the group. The collections of the Natural History Museums in Paris and London, and the Museum and Institute of Zoology in Warsaw house all types of this group and they were studied to present a revision which is intended to provide comprehensive information on Javanese species.

## Material and methods

Species delineation and diagnoses are based on the male adult semaphoronts if a male is available. The unique types represented by females represent a problem, as assignment of conspecific males and females is difficult. Diagnoses of female type specimens are based on the morphology of the ovipositor, as the shape of the antennae and relative size of the eyes are uniform.

Male and female genitalia were studied. Dry mounted specimens were transferred to 50% ethanol and apical parts of abdomens were shortly kept in hot 10% KOH to clean them of muscles and fat bodies. Photographs of diagnostic characters and measurements were taken using an Olympus SZX-16 microscope. The following measurements were taken: BL – body length; HW – width at the humeri; PW – pronotal width, measured at the base; PL – pronotal length at midline; Edist – minimum frontal distance between eyes; Ediam – maximum eye diameter in the lateral view.

*Depositories*: BMNH *–* Natural History Museum, London; KMCT *–* Kiyoshi Matsuda Collection, Takarazuka city; LMBC *–* Dept. of Zoology, Palacky University, Olomouc; MHNP *–* Museum d’histoire naturelle, Paris; MIZW *–* Museum and Institute of Zoology PAN, Warszawa.

## Taxonomy

### Cautires

Waterhouse, 1879

http://species-id.net/wiki/Cautires

#### Type species.

*Lycus* (gen. 22) *excellens* Waterhouse, 1878; Bourgeois 1891: 345, by subsequent designation.

#### Differential diagnosis of the *Cautires obsoletus* species group.

**Figures 1–11.**
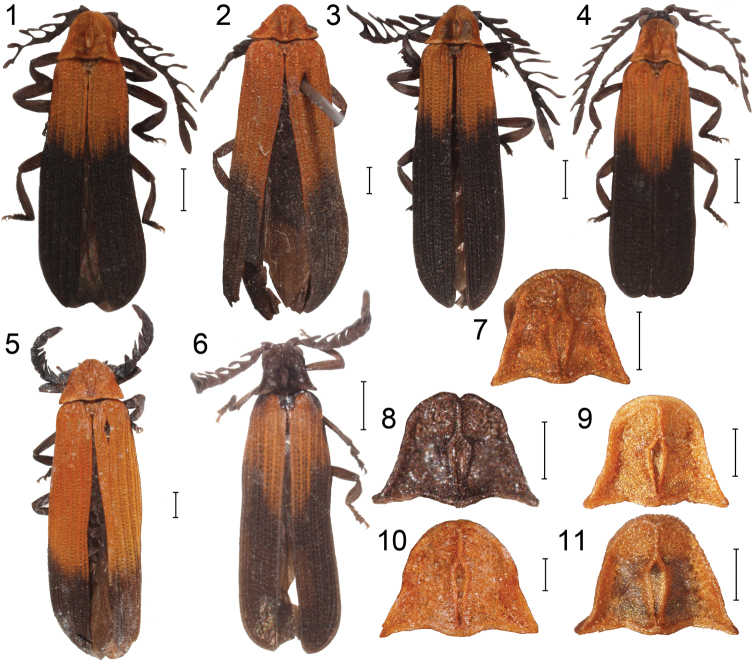
General appearance: **1** *Cautires taoi* sp. n. **2** *Cautires singularithorax* Pic, holotype **3** *Cautires sukosarensis* sp. n. **4** *Cautires walteri* sp. n. **5** *Cautires apicalis* Pic, holotype sp. **6** *Cautires obsoletus* Waterhouse. Pronotum: **7** *Cautires walteri* sp. n. **8** *Cautires obsoletus* Waterhouse **9** *Cautires taoi* sp. n. **10** *Cautires apicalis* Pic, holotypesp. **11** *Cautires sukosarensis* sp. n. Scale bars Figs 1–6 = 1.0 mm, Figs 7–11 = 0.5 mm.

**Figures 12–23.**
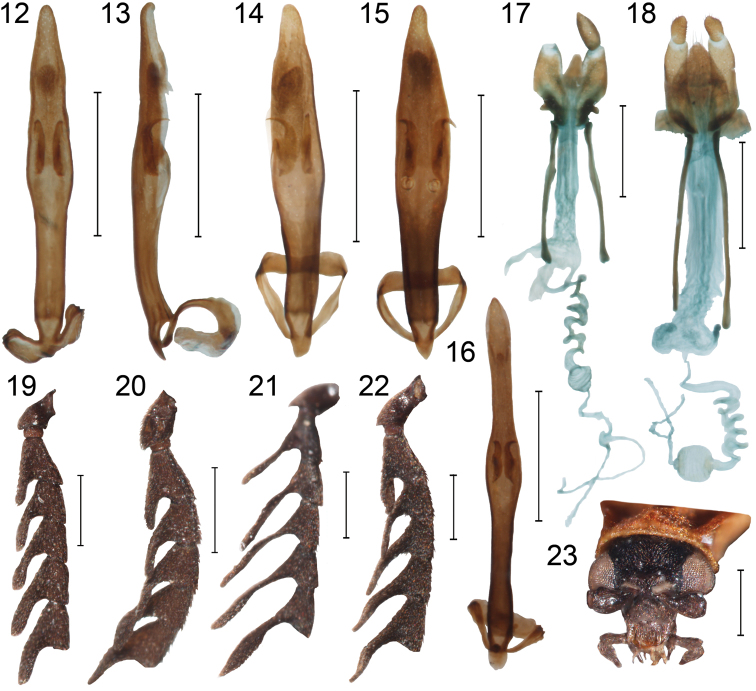
Male genitalia: **12, 13** *Cautires obsoletus* Waterhouse **14** *Cautires walteri* sp. n. **15** *Cautires taoi* sp. n. **16** *Cautires sukosarensis* sp. n. Female genitalia: **17** *Cautires singularithorax* Pic, holotype **18** *Cautires apicalis* Pic, holotype. Male basal antennomeres **19** *Cautires sukosarensis* sp. n. **20** *Cautires obsoletus Waterhouse* **21** *Cautires sukosarensis* sp. n. **22** *Cautires taoi* sp. n. **23** *Cautires walteri* sp. n., head frontally. Scale bars = 0.5 mm.

*Cautires* belongs to the tribe Metriorrhynchini, which is easily recognizable by well-developed pronotal and elytral costae, a circular phallobase and unpaired gland in the vagina (Bocak 2002).All species classified in the *Cautires obsoletus* group share principal diagnostic characters with other *Cautires*: a medium sized, flattened, feebly sclerotized body, flabellate antennae in males which are serrate in females, four primary and five secondary longitudinal costae in the elytra, and a lanceolate phallus with membranous internal sac bearing two sickle-shaped thorns at its base (Figs 1–23). The species groupis defined by the presence of a single median areola in the pronotum and it differs from the *Cautires pauper* species group in the obtuse frontal angles resulting in the triangular shape of the pronotum, and a slender phallus (Figs 7–16). The morphology of *Cautires* was described and illustrated in detail by Dudkova and Bocak (2010).

#### Distribution and biology.

The low dispersal propensity of metriorrhynchine net-winged beetles results in small ranges and effectiveness of barriers, which are crossed easily by other beetles (Kubecek et al. 2011). Adults of net-winged beetles usually remain under the canopy of the tropical rain forest and do not fly in open habitat (Bocak 2002). Extensive studies of South East Asian *Cautires* and other Metriorrhynchini revealed that each species is typically restricted to a single island, and that these beetles very seldom have extensive ranges (e.g. Dudkova and Bocak 2010, Weiszenstein and Bocak 2011). Although Kleine (1933) recorded several species of *Cautires* from two or more Great Sunda islands, the study of principal collections and extensive collecting activity in Sumatra and Borneo revealed that all Javanese species treated here are endemic to Java and do not occur in the other Great Sunda islands. So, for example, due to high similarity, *Cautires apicalis* described from Java has been for long time kept in synonymy of *Cautires corporaali* from Northern Sumatra and is reinstated as a valid species.

The second potential reason for the high level of endemism in net-winged beetles is the role of aposematic coloration. The majority of Metriorrhynchini in the Oriental Region are aposematically colored and the Javanese *Cautires* species are no exception (Figs 1–6). The similar orange and black pattern is known also from higher mountain regions of Sumatra, e.g. Gunung Kerinci, Gunung Merapi and volcanoes in the vicinity of Brastagi. These aposematic patterns are limited to higher mountain habitats and the lower areas are inhabited by differently colored species. The role of color patterns as a factor limiting dispersal was discussed by Bocak and Yagi (2010).

#### Key to the *Cautires obsoletus* species group of Java

##### 

**Table:** 

1	Pronotum dark brown to black (Figs 6, 8)	*Cautires obsoletus* (Waterhouse)
–	Pronotum brightly colored, similarly to basal part of elytra (Figs 1–5, 7, 9–11)	2
2	Body large, over 10 mm, orange part of elytra reaching over half of elytral length	3
–	Body small, less than 9 mm, orange part of elytra reaching less than half of elytral length	4
3	About apical quarter of elytra dark colored (Fig. 5), female genitalia with valvifers 2.1 times the length of coxites (Fig. 18)	*Cautires apicalis* (Pic)
–	Almost half of elytra dark colored (Fig. 2), regularly, female genitalia with valvifers 1.75 times the length of coxites (Fig. 17)	*Cautires singularithorax* (Pic)
4	Male eyes large, their frontal minimum distance 0.87 times maximum diameter in lateral view	*Cautires taoi* Bocak, sp. n.
–	Male eyes small, their frontal minimum distance more than 1.20 times maximum diameter in lateral view	5
5	Phallus very slender, parallel-sided in most of its length, widened at middle, sclerotized spines of internal sac small, phallus about 8.8 times longer than spines of internal sac (Fig. 16)	*Cautires sukosarensis* Bocak, sp. n.
–	Phallus moderately slender, narrower towards base and apex, widest at middle of its length, sclerotized spines of internal sac small, phallus about 6.2 times longer than spines of internal sac (Fig. 14)	*Cautires walteri* Bocak, sp. n.

### Cautires apicalis

(Pic, 1925b) stat. n.

http://species-id.net/wiki/Cautires_apicalis

Figs 5, 10 , 18

Bulenides apicalis Pic 1925b: 9.
Cautires apicalis (Pic 1925b): Dudkova and Bocak 2010: 41.

#### Type material.

Female, holotype. [Indonesia] Java occident. Pengalengan, 4000’, 1893, H. Fruhstorfer (MHNP).

#### Differential diagnosis.

*Cautires apicalis* differs from the similar Javanese species *Cautires singularithorax* in the much smaller extent of the dark part of the elytra and in the shape of the ovipositor, which has valvifers more than two times longer than the coxites (Figs 2, 5, 18). Only female specimen is available and we do not have any information on male characters.

#### Redescription.

Female. Body medium-sized, dorso-ventrally flattened, slender. Head, body, posterior third of elytra and appendages black, pronotum and basal two thirds of elytra orange, pronotum and elytra covered with dense orange pubescence (Figs 5, 10). Head small, partly hidden in pronotum, clypeus slightly concave, labrum simply rounded, mandibles slender, strongly curved, maxillary palpi slender, apical palpomere pointed, labial palpi similar in shape. Eyes small, hemispherically prominent, their frontal interocular distance 1.50 times eye diameter. Antennae acutely serrate, covered with short, dark colored setae. Pronotum flat, slightly transverse, 1.32 times wider at base than length at midline; frontal margin concave; lateral margins slightly elevated, posterior angles acutely projected, pronotum with slender median areola, attached to middle of basal margin of pronotum, connected to anterior margin by keel occupying one third of midline, lateral keels absent (Fig. 10). Elytra flat, with separately rounded apexes and well developed four primary longitudinal costae; secondary costae very weak, cells irregular, mostly quadrate. Legs laterally flattened, covered with dark colored setae. Ovipositor with valvifers 2.1 times length of coxites (Fig. 18). Male unknown.

#### Measurements.

BL 11.9 mm, PL 1.84 mm, PW 2.42 mm, HW 2.72 mm, Edist 0.69 mm, Ediam 0.46 mm.

#### Distribution.

*Cautires apicalis* is known only from Western Java.

#### Remarks.

Kleine (1933) listed *Cautires apicalis* as a junior synonym of *Cautires corporaali* Pic, 1921 from Sumatra, but the study of the holotypes revealed that these species although similar in the color patterns differ in the shape of the pronotum, body size, and female genitalia. Therefore the name *Cautires apicalis* is removed from synonymy and reinstated as a valid name.

Two large bodied species were described by M. Pic from Western Java, both of them from a single female specimen. Although one male specimen collected in the same region was available for study, it is impossible to assign the name based on female to a male without further information. Therefore, holotypes of *Cautires apicalis* and *Cautires singularithorax* are redescribed and illustrated here. More extensive material is necessary for the definitive delineation of these species.

### Cautires obsoletus

(Waterhouse, 1878)

http://species-id.net/wiki/Cautires_obsoletus

Figs 6, 8 , 12–13, 20

Bulenides obsoletus Waterhouse 1878: 109.
Cautires obsoletus (Waterhouse 1878): Dudkova and Bocak 2010: 43.
Bulenides lineatus Pic 1921: 8.
Cautires lineatus (Pic 1921): Dudkova and Bocak: 42 (a junior secondary homonym of *Cautires lineatus* (Hope in Gray 1831), syn. n.
Cautires fruhstorferi Bocak and Dudkova 2010: 42 (a replacement name for *Bulenides lineatus*), syn. n.
Bulenides javanicus Bourgeois 1883: 439.
Cautires javanicus (Bourgeois 1883): Dudkova and Bocak 2010: 42, syn. n.
Bulenides inhumeralis Pic 1921: 7.
Cautires inhumeralis (Pic 1921): Dudkova and Bocak 2010: 42, syn. n.
Bulenides nigromaculatus Pic 1925a: 7.
Cautires nigromaculatus (Pic 1925a): Dudkova and Bocak 2010: 43, syn. n.
Bulenides pudicus Kleine 1931: 257.
Cautires pudicus (Kleine 1931): Dudkova and Bocak 2010: 43, syn. n.

#### Type material.

Female, holotype of *Bulenides obsoletus*. [Indonesia] Java (without further data, BMNH). Female, holotype of *Bulenides lineatus*. [Indonesia] Java occident., Sukabumi, 2000’, 1893, H. Fruhstorfer (MHNP). Male, holotype of *Bulenides javanicus*. [Indonesia, Java] Giava, Tcibodas, Ott. 1874, O. Beccari (MHNP). Male, holotype of *Bulenides inhumeralis*. [Indonesia] Bogor, 1000’, v–vi 96, I. Z. Kannegieter (MHNP). Male, holotype of *Bulenides nigromaculatus*. [Indonesia] Java occident., Sukabumi 2000’, H. Fruhstorfer (MHNP). Male, holotype of *Bulenides pudicus*. [Indonesia] Toegoe, West-Jawa-Pasteur (without further data, MIZW).

#### Differential diagnosis.

*Cautires obsoletus* differs from the other Javanese species in the black pronotum (Fig. 8) and a very oblique border between the bright and dark parts of elytra (Fig. 6). The basal part of elytra is brown to reddish brown and differs from the brightly orange coloration of the other species in Java.

#### Redescription

**.**Male. Body small to medium-sized, dorso-ventrally flattened, slender; head, body, posterior half of elytra and appendages dark-brown to black; basal half of elytra brown to reddish brown (Fig. 6), body covered with dense pubescence. Head small, partly hidden in pronotum, clypeus slightly concave, labrum simply rounded, mandibles slender, strongly curved, maxillary palpi slender, apical palpomere pointed; labial palpi similar in shape. Eyes small, hemispherically prominent, their frontal interocular distance 1.15 times eye diameter. Antennae shortly flabellate, 11-segmented, covered with short, dark colored setae (Fig. 20). Pronotum flat, only slightly transverse, 1.15 times wider at base than length at midline; frontal margin projected forward; lateral margins slightly elevated, concave, posterior angles acutely projected, pronotum with moderately wide median areola, attached to middle of basal margin of pronotum, connected to anterior margin by keel occupying one third of midline, lateral keels absent (Fig. 8). Elytra flat, with well developed four primary longitudinal costae; secondary costae considerably weaker, cells regular, mostly slightly longitudinal. Legs laterally flattened, covered with dark colored setae. Male genitalia with phallus widest at midlength and gradually narrowed to apex (Figs 12–13). Female. Body medium-sized, antennae serrate, ovipositor with short valvifers.

#### Measurements.

BL 6.45 mm, PL 1.01 mm, PW 1.36 mm, HW 1.47 mm, Edist 0.51 mm, Ediam 0.44 mm.

#### Distribution.

*Cautires obsoletus* is known only from several localities in Western Java.

#### Material examined.

2 males, 1 female, Indonesia, W. Java, Puncak Pass nr Bogor, 23. Mar. 1992, H. Arimoto, lgt.; male, [Indonesia] West Java, Puncak Pass, 22. Mar. 1993, Y. Miyake leg. (KMTC, LMBC).

#### Remark.

The holotype of *Cautires obsoletus* is a strongly damaged female with only a part of one elytron preserved. The basal part of the elytron is testaceous and the loss of reddish coloration may be caused by long-term exposure to light. The holotype of *Cautires lineatus* is also a female and it differs only in the darker hue of the bright part of the elytra, but it resembles the holotype of *Cautires obsoletus* in its body shape. The female genitalia of all available specimens are very similar.Holotypes of four other species are males and they do not differ in comparable characters such as body shape and color pattern. Therefore, all these species are considered junior subjective synonyms of *Cautires obsoletus*.

### Cautires singularithorax

(Pic, 1925a)

http://species-id.net/wiki/Cautires_singularithorax

Figs 2 , 17

Bulenides singularithorax Pic 1925a: 7.
Cautires singularithorax (Pic 1925a): Dudkova and Bocak 2010: 43.

#### Type material.

Female, holotype. [Indonesia] Coll. Dr. H. J. Veth, P. J. Sijthoff, Java, Preanger (MHNP).

#### Differential diagnosis.

*Cautires singularithorax* resemble *Cautires apicalis* in the body size and color pattern (Figs 2, 5). These species differ in the extent of the bright part of the elytra, which is much smaller in *Cautires singularithorax* and in V-shaped border between bright and dark part in *Cautires singularithorax* and almost transverse border in *Cautires apicalis*. Female genitalia of both species differ in the relative length of valvifers, those of *Cautires singularithorax* are stout and about 1.8 times longer than coxites (Figs 17, 18). *Cautires singularithorax* has not been found in the available recently collected material and only unique female specimen is known and we do not have any information on male characters.

#### Redescription.

Female. Body medium-sized, dorso-ventrally flattened, slender; head, body, posterior half of elytra and appendages dark-brown to black; pronotum and basal half of elytra orange, pronotum and elytra covered with dense orange pubescence. Head small, partly hidden in pronotum, clypeus slightly concave, labrum simply rounded, mandibles slender, strongly curved apically, maxillary with apical palpomere pointed; labial palpi similar in shape. Eyes small, hemispherically prominent, their frontal interocular distance 1.52 times eye diameter. Antennae acutely serrate, covered with short, dark colored setae. Pronotum flat, transverse, 1.45 times wider at base than length at midline; frontal margin concave; lateral margins slightly elevated, posterior angles very acutely projected, pronotum with median areola, areola widest anteriorly and attached to middle of basal margin, connected to anterior margin by keel occupying one third of midline. Elytra flat, with well developed four primary longitudinal costae; secondary costae considerably weaker, often interrupted, cells irregular, often inconspicuous, mostly quadrate. Legs laterally flattened, covered with dark colored setae. Ovipositor with valvifers 1.75 times length of coxites (Fig. 25). Male unknown.

#### Measurements.

BL 11.8 mm, PL 1.76 mm, PW 2.56 mm, HW 3.04 mm, Edist 0.74 mm, Ediam 0.48 mm, length of valvifer 0.78 mm, length of coxite 0.44 mm.

#### Distribution.

The species is known only in the type specimen from Java.

### Cautires taoi

Bocak sp. n.

urn:lsid:zoobank.org:act:3B3082A2-0C0E-42C8-BFE9-66B92DF0D789

http://species-id.net/wiki/Cautires_taoi

Figs 1, 9 , 15, 22

#### Type material. 

Male, holotype. Java, 13–14 km from Sukosari, 25. May 1982, M. Tao (KMTC). Paratypes. 2 males, data same as for holotype, 26. May 1982; 2 males, Java, Mt. Idjen, 15.–16. May 1982; female, E Java, Ijen, Jamba, 18. Apr. 1981, H. Detani leg.; female, E Jawa, Jambu Lijen, Banyuwangi, 12. Aug. 1986, T. Ito leg. (KMTC, LMBC).

#### Etymology.

The specific epithet is a patronym in honour of Mr M. Tao (Japan), the collector of the species.

#### Differential diagnosis.

*Cautires taoi* belongs along with *Cautires sukosarensis* and *Cautires walteri* to a group of the small-bodied, aposematically colored species with brightly colored pronotum and basal half of elytra (Figs 1, 3–4). This species differs in the large eyes, which are the largest within Javanese species, and the shape of male genitalia (Fig. 15).

#### Description.

Male. Body small-sized, dorso-ventrally flattened, slender, body, posterior half of elytra and appendages dark-brown to black (Fig. 1); pronotum and basal half of elytra bright orange red, pronotum and elytra covered with dense orange pubescence (Fig. 1). Head small, partly hidden in pronotum, clypeus slightly concave, labrum simply rounded, mandibles strongly curved apically, maxillary palpi with apical palpomere pointed; labial palpi similar in shape. Eyes large, hemispherically prominent, their frontal interocular distance 0.87 times eye diameter. Antennae shortly flabellate, covered with short, dark colored setae (Fig. 22). Pronotum flat, slightly transverse, 1.35 times wider at base than length at midline; frontal margin projected forward; lateral margins slightly elevated, convex, posterior angles acutely projected, pronotum with moderately robust median areola, attached to middle of basal margin of pronotum, connected to anterior margin by keel occupying one third of midline, lateral keels absent, anterior and lateral part of pronotum with fine, inconspicuous punctures (Fig. 9). Elytra flat, with well developed four primary longitudinal costae; secondary costae weaker, cells regular, tiny, mostly longitudinal. Legs laterally flattened, covered with dark colored setae. Phallus moderately robust, simple, almost parallel-sided (Fig. 15). Female slightly larger, similar in body coloration, antennae serrate. Valvifers 1.3 times length of coxites.

#### Measurements.

BL 7.15 mm, PL 1.17 mm, PW 1.54 mm, HW 1.69 mm, Edist 0.50 mm, Ediam 0.57 mm.

#### Distribution.

*Cautires taoi* is known at present only in the type series from Java.

### Cautires sukosarensis

Bocak sp. n.

urn:lsid:zoobank.org:act:2E02589C-484A-4078-98BD-8B52CFB151FD

http://species-id.net/wiki/Cautires_sukosarensis

Figs 3, 11 , 16, 21

#### Type material.

Male, holotype. Java, 14–16 km from Sukosari, 22. May 1982, M. Tao (KMTC). Paratypes, male, female. Java, 14 km from Sukosari, 23. May 1982, 25. May 1982, M. Tao (KMTC, LMBC).

#### Etymology.

The specific epithet refers to the type locality.

#### Differential diagnosis.

*Cautires sukosarensis* belongs along with *Cautires walteri* and *Cautires taoi* to a group of the small bodied aposematically colored species with brightly colored pronotum and basal half of elytra (Figs 1, 3, 4). This species resembles *Cautires walteri* in relatively small eyes and differs from other species in the extremely slender and long phallus and long antennal lamellae (Figs 16, 21). Male genitalia are similar to those of *Cautires bolavensis* Dudkova & Bocak, 2010 from Laos, but these species differ in the coloration and the size of eyes. Similarity of genitalia may indicate the close relationships of vicariant species from Laos and Java as reported by Bocak and Yagi (2010).

#### Description.

Male. Body small-sized, dorso-ventrally flattened, moderately slender; body, posterior half of elytra and appendages dark-brown to black; pronotum and basal half of elytra bright orange red, pronotum and elytra covered with dense orange pubescence (Fig. 3). Head small, clypeus slightly concave, labrum simply rounded, mandibles strongly curved apically, palpi with apical palpomeres pointed. Eyes small, hemispherically prominent, their frontal interocular distance 1.28 times eye diameter. Antennae shortly flabellate, 11-segmented, covered with short, dark colored setae (Fig. 21). Pronotum flat, transverse, 1.39 times wider at base than length at midline; frontal margin projected forward; lateral margins slightly elevated, convex, posterior angles acutely projected, pronotum with moderately robust median areola, attached to middle of basal margin of pronotum, connected to anterior margin by keel occupying one third of midline, lateral keels absent (Fig. 11). Elytra flat, with separately rounded apexes and well developed four primary longitudinal costae; secondary costae weak, cells regular, tiny, mostly quadrate. Legs laterally flattened, covered with dark colored setae. Phallus very slender, long, widest at midlength (Fig. 19). Female slightly larger, similar in body coloration, antennae serrate. Valvifers 1.55 times length of coxites.

#### Measurements.

BL 8.05 mm, PL 1.18 mm, PW 1.64 mm, HW 1.89 mm, Edist 0.55 mm, Ediam 0.43 mm.

#### Distribution.

*Cautires sukosarensis* is known at present only in the type series from Eastern Java.

### Cautires walteri

Bocak sp. n.

urn:lsid:zoobank.org:act:F2656D4E-5E00-463E-9193-9C10BBF546AB

http://species-id.net/wiki/Cautires_walteri

Figs 4, 7 , 14, 19, 23

#### Type material.

Holotype. Male, Java, Rancabali, 45 km S of Bandung, 1700 m, 12. Oct. 2002, Bolm lgt. (LMBC). Paratypes. 2 males, data same as for holotype (KMTC, LMBC).

#### Etymology.

The specific epithet is a patronym in honour of the late Dr Walter Wittmer (Basel).

#### Differential diagnosis.

*Cautires walteri* resemble *Cautires sukosarensis* in the relatively small eyes and these species differ in the shape of body and male genitalia. *Cautires walteri* is characterized by the slender, small body and moderately robust phallus (Figs 1, 14).

#### Description.

Male. Body small-sized, dorso-ventrally flattened, slender; body, posterior half of elytra and appendages dark-brown to black; pronotum and basal half of elytra bright orange red, pronotum and elytra covered with dense orange pubescence (Fig. 4). Head small, clypeus slightly concave, labrum simply rounded, mandibles slender, strongly curved, apical palpomeres pointed (Fig. 23). Eyes small, hemispherically prominent, their frontal interocular distance 1.25 times eye diameter. Antennae shortly flabellate, 11-segmented, covered with short, dark colored setae (Fig. 19). Pronotum flat, transverse, 1.19 times wider at base than length at midline; frontal margin projected forward; lateral margins slightly elevated, convex, posterior angles acutely projected, pronotum with moderately robust median areola, attached to middle of basal margin of pronotum, connected to anterior margin by keel occupying one third of midline, lateral keels absent. Elytra flat, with well developed four primary longitudinal costae; secondary costae considerably weaker, cells regular, tiny, mostly quadrate. Legs laterally flattened, covered with dark colored setae. Phallus moderately robust, widest at midlength (Fig. 14). Female unknown.

#### Measurements.

BL 6.65 mm, PL 1.01 mm, PW 1.20 mm, HW 1.50 mm, Edist 0.50 mm, Ediam 0.40 mm.

#### Distribution.

*Cautires walteri* is known only in the type series from Western Java.

## Supplementary Material

XML Treatment for Cautires

XML Treatment for Cautires apicalis

XML Treatment for Cautires obsoletus

XML Treatment for Cautires singularithorax

XML Treatment for Cautires taoi

XML Treatment for Cautires sukosarensis

XML Treatment for Cautires walteri
